# Current Status and Progress of Targeted and Immunotherapy for DSRCT

**DOI:** 10.3390/cancers18111711

**Published:** 2026-05-24

**Authors:** Tian Wei, Qidi Zhao, Yan Li

**Affiliations:** Department of Peritoneal Oncology, Beijing Tsinghua Changgung Hospital, School of Clinical Medicine, Tsinghua Medicine, Tsinghua University, Beijing 102218, China; weit23@mails.tsinghua.edu.cn (T.W.); zqd23@mails.tsinghua.edu.cn (Q.Z.)

**Keywords:** desmoplastic small round cell tumor (DSRCT), EWSR1-WT1, targeted therapy, immunotherapy, DNA damage response (DDR)

## Abstract

Desmoplastic small round cell tumor (DSRCT) is a rare and highly malignant tumor that mostly occurs in young males. Due to its extremely strong invasiveness and poor prognosis, the treatment of DSRCT remains a major challenge in current medical research. The comprehensive treatment strategy based on surgery, combined with chemotherapy, targeted therapy, immunotherapy has become a clinical consensus. This review summarizes the main pathogenic mechanisms of DSRCT, as well as the targets involved in treatment and their applications, including targeted therapy targets (PDGF, VEGFR, FGFR4, IGF1R, HER2, c-KIT, mTOR, AR), immunotherapy targets (PD-1, PD-L1, B7H3, GD2), and treatments related to DNA damage response. Studies have shown that treatments targeting specific targets can inhibit tumor progression and prolong patient survival to a certain extent, but the efficacy has individual differences and is still limited. Therefore, future research still needs to further explore the molecular mechanism of DSRCT and discover more accurate and effective therapeutic targets.

## Simple Summary

Desmoplastic small round cell tumor (DSRCT) is a rare, highly aggressive soft tissue malignancy with poor prognosis and limited standardized treatment options. Current mainstream therapies including surgery, chemotherapy, targeted therapy and immunotherapy cannot achieve durable disease control for most patients. This review systematically summarizes the pathogenic mechanism of DSRCT driven by EWSR1-WT1 fusion and the latest research progress of targeted and immunotherapeutic targets, and clarifies the clinical evidence level of different therapies. The study aims to provide systematic evidence and feasible research directions for optimizing individualized treatment strategies and developing novel therapeutic targets for DSRCT.

## 1. Introduction

Desmoplastic small round cell tumor (DSRCT) is an extremely rare malignant soft tissue neoplasm with an incidence of approximately one in five million individuals [1]. In 1989, Gerald and Rosai [2], Ordóñez [3] and Gaffney et al. [4] successively reported cases of this rare tumor. In 1991 [5], by analyzing and summarizing 19 collected clinical cases, Gerald and Rosai first clarified the independent pathological properties of DSRCT and systematically summarized its clinical and pathological features. They formally coined the disease nomenclature and established standardized diagnostic criteria for this entity. To date, this seminal work has been universally acknowledged as the fundamental basis for the academic definition and pathological classification of DSRCT worldwide. DSRCT predominantly affects young males, with a male-to-female ratio of approximately 4–5:1 [6,7,8], and a peak age of onset between 20 and 30 years [9,10,11,12]. The tumor typically arises from abdominopelvic soft tissues and exhibits highly aggressive behavior, with frequent metastatic spread to sites including the colorectum, small intestine, ovary, bladder, and liver [13]. Extra-abdominal involvement may also occur in the ovaries, paratesticular region, bone, soft tissues, and pleura, although such presentations are relatively uncommon [14].

In the early stages of disease, patients are often asymptomatic or present only with non-specific symptoms such as mild abdominal pain, abdominal distension, or palpable abdominal masses [15]; some patients also develop ascites [16,17]. Due to its non-specific early clinical manifestations, DSRCT is prone to misdiagnosis or delayed diagnosis, and most patients are already at an advanced stage with metastatic disease at the time of confirmation. DSRCT carries an extremely poor prognosis: despite comprehensive therapeutic strategies including surgery, chemotherapy, targeted therapy, and radiotherapy, the 5-year survival rate remains only approximately 15–25% [18,19,20,21,22,23].

The alkylating agent-based P6 regimen (cyclophosphamide, doxorubicin, vincristine, ifosfamide, etoposide) [24] has become the mainstream chemotherapy regimen for DSRCT. This regimen was originally adapted from that used for Ewing’s sarcoma (ES), given the similarities between DSRCT and ES. Mechanistically, the key driver of ES is the is the gene fusion between Ewing sarcoma RNA-binding protein 1 (EWSR1) and Friend leukemia integration 1 (FLI1), while DSRCT is driven by the fusion of EWSR1 and Wilms tumor 1 (WT1)—both share the EWSR1 gene as one component of the fusion. Furthermore, studies have shown partial overlap in the downstream gene regulatory pathways of EWSR1-WT1 and EWSR1-FLI1 [25,26], with ERG identified as a potential common target gene that regulates tumor progression toward an ES-like phenotype [27]. Gedminas et al. [25] demonstrated that silencing EWSR1-WT1 in DSRCT cell lines led to downregulation of multiple EWSR1-FLI1 target genes; silencing ERG resulted in significant loss of tumor cell viability and increased apoptosis, similar to the effects of EWSR1-WT1 silencing. Gene functional enrichment analysis further suggested that EWSR1-FLI1 and EWSR1-WT1 may share common mechanisms of gene expression dysregulation, further supporting the similarities between DSRCT and ES. Due to the limited overall efficacy of conventional chemotherapy, novel regimens are being actively explored, including the VAIA regimen (ifosfamide, vincristine, doxorubicin, actinomycin D) [16] and the VIT regimen (vincristine, irinotecan, temozolamide) [28], both of which have shown promising preliminary results. Additionally, radiotherapy, including whole abdominopelvic radiotherapy (WAPT) and intensity-modulated radiotherapy, can be used as an adjuvant treatment [29].

Histologically, DSRCT is characterized by small, round, basophilic cells arranged in variably sized nests, separated by prominent proliferative fibrous stroma. Immunohistochemical analysis reveals a multiphenotypic profile, with concurrent expression of epithelial, mesenchymal, myogenic, and neurogenic markers [5,9,30,31]. Among these, co-expression of cytokeratin (CK) and desmin is relatively specific for DSRCT and aids in its differential diagnosis from other small round cell tumors (SRCTs), such as ES and peripheral primitive neuroectodermal tumor (PNET). This multiphenotypic nature suggests that DSRCT may originate from progenitor cells with multi-differentiation potential [32].

Over recent decades, extensive efforts have been made to elucidate the pathogenesis of DSRCT. Chromosomal translocation leading to the fusion of EWSR1 and WT1 genes is widely recognized as the driver and signature genetic alteration of DSRCT, although rare instances have been documented in other tumor types [33]. This review summarizes the current understanding of the pathogenic mechanisms and potential therapeutic targets of DSRCT, with the aim of advancing translational research and clinical management of this devastating malignancy.

## 2. Literature Search Strategy

A systematic literature search was performed to collect eligible studies on DSRCT targeted and immunotherapy. The searched databases included PubMed, Web of Science and ClinicalTrials.gov, with the final search updated in March 2026. Core search keywords were DSRCT, desmoplastic small round cell tumor, and EWSR1-WT1. The inclusion criteria prioritized human clinical studies (prospective trials and retrospective cohorts), with well-verified preclinical mechanism studies included to supplement pathogenic and therapeutic molecular mechanisms, and relevant high-quality reviews incorporated to synthesize evidence and ensure comprehensiveness.

## 3. Pathogenesis of DSRCT

**Figure 1:**
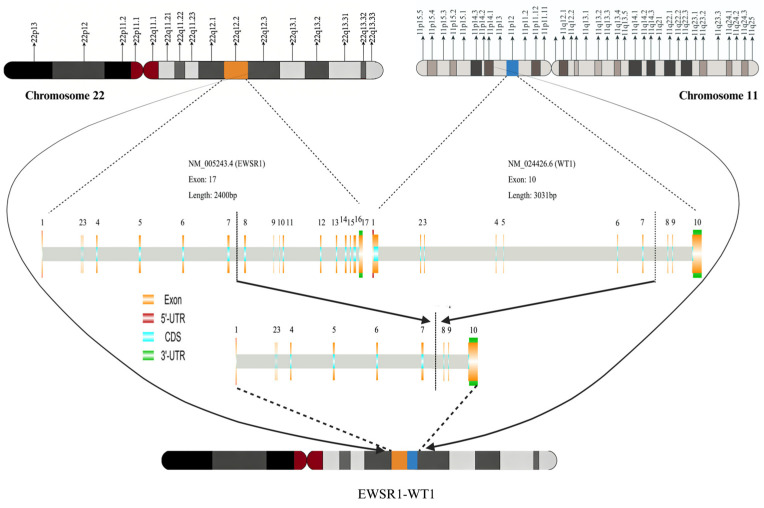
Schematic representation of the chromosomal translocation and generation of the EWSR1-WT1 fusion gene (IBS 2.0 is used: an upgraded illustrator for the visualization of biological sequences). EWSR1: Ewing sarcoma RNA-binding protein 1; WT1: Wilms tumor 1.

The fundamental mechanism underlying DSRCT development lies in genetic alterations. In 1992, Sawyer et al. [34] first described the characteristic chromosomal translocation: reciprocal translocation between WT1 (located at chromosome 11p13) and EWSR1 (located at chromosome 22q12), designated as t(11;22)(p13;q12) (Figure 1). In 1994, Ladanyi and Gerald experimentally confirmed the presence of this EWSR1-WT1 fusion gene in DSRCT as the pathognomonic molecular event [35]. Subsequently, they further characterized the genomic breakpoint distribution and transcript features of the fusion gene [36], establishing DSRCT as the first human neoplasm associated with WT1 translocation and the third disease driven by EWSR1 rearrangement.

The major breakpoint cluster region of EWSR1 (NM_005243) is localized to exons 7–10, with most breakpoints occurring between exons 7 and 8. In contrast, the majority of WT1 breakpoints are concentrated in the region between exons 7 and 8 [37,38,39,40]. We hereby discuss the features of the two genes separately to facilitate a better understanding of the structure and functional properties of fusion genes.

WT1 was initially recognized as a tumor suppressor, as its inactivation was directly implicated in the pathogenesis of Wilms tumor [41]. Subsequent studies have established that WT1 acts as an indispensable regulator of embryonic development, with critical roles in the ontogeny of the genitourinary system, spleen, and mesothelium [42]. The WT1 protein contains an *N*-terminal region enriched in proline and glutamine residues that mediates core transcriptional regulatory functions, and a *C*-terminal domain primarily composed of four Cys2-His2 (C2H2) zinc fingers (ZNFs). This structural arrangement confers robust sequence-specific DNA-binding activity, as well as weak RNA-binding capacity. Specifically, zinc fingers 3–4 (especially ZNF4) govern high-affinity DNA binding, whereas ZNF1 is strictly required for RNA interaction [43].

WT1 encodes two major alternatively spliced isoforms, namely WT1(+KTS) and WT1(-KTS), which differ by the insertion of a lysine-threonine-serine (KTS) tripeptide between ZNF3 and ZNF4 in the former isoform. Mechanistic studies have demonstrated that this KTS insertion sterically impairs the function of ZNF4, reducing the DNA-binding affinity of WT1 by more than 10-fold, thereby endowing the two isoforms with distinct biological roles. Under physiological conditions, WT1(-KTS) is diffusely distributed within the nucleus, colocalizes with transcription factors, and directly binds target DNA sequences to modulate downstream gene expression. In contrast, WT1(+KTS) localizes to nuclear speckles and colocalizes with nuclear splicing factors. Although WT1(+KTS) is more abundant in vivo, it rarely induces overt cellular phenotypes or alters target gene expression; instead, it is thought to bind mRNA and participate in post-transcriptional modifications [38,44]. The two isoforms are equally essential for normal human growth and development, and maintain a stable physiological ratio of approximately 2:1 (+KTS: €KTS) [45]. Disruption of this balanced ratio leads to a spectrum of developmental disorders, such as Frasier syndrome (ratio is 0.5), which is characterized by severe genitourinary defects.

**Figure 2:**
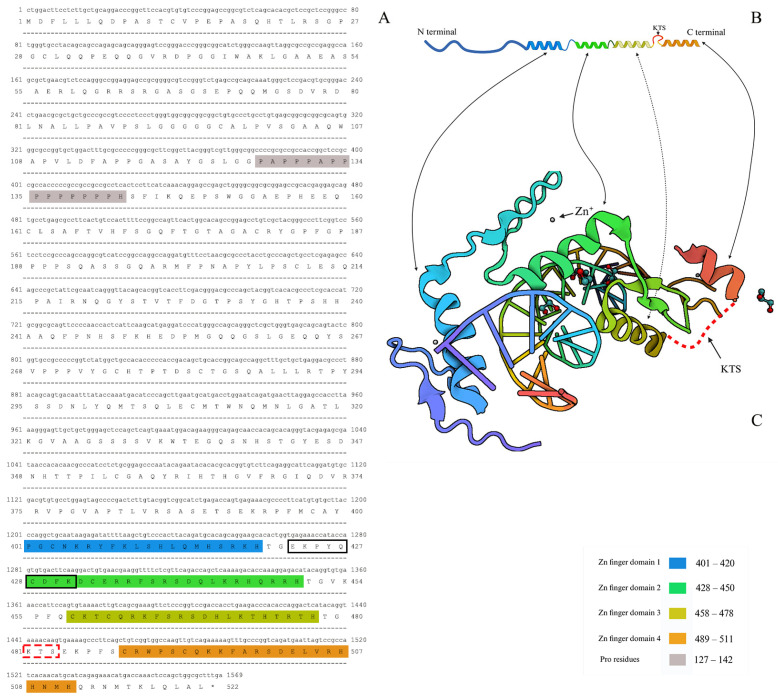
(**A**): Nucleotide sequence of the WT1 transcript (NM_024426.6) and corresponding amino acid sequence (NP_077744.4). * means that this codon does not encode an amino acid. (**B**): Schematic diagram of the WT1 domain structure. The KTS insertion site is highlighted in the red dashed box; the frequent breakpoint region is marked in the black box. (**C**): Ribbon diagram of the WT1 tertiary structure (PDB ID: 6BLW).

WT1 is prone to genomic cleavage, and its fusion with the EWSR1 gene represents the hallmark oncogenic event in DSRCT. A frequent breakpoint cluster is located within the peptide sequence SEKPYQCDFK, positioned between ZNF1 and ZNF2 (Figure 2) [46].

**Figure 3:**
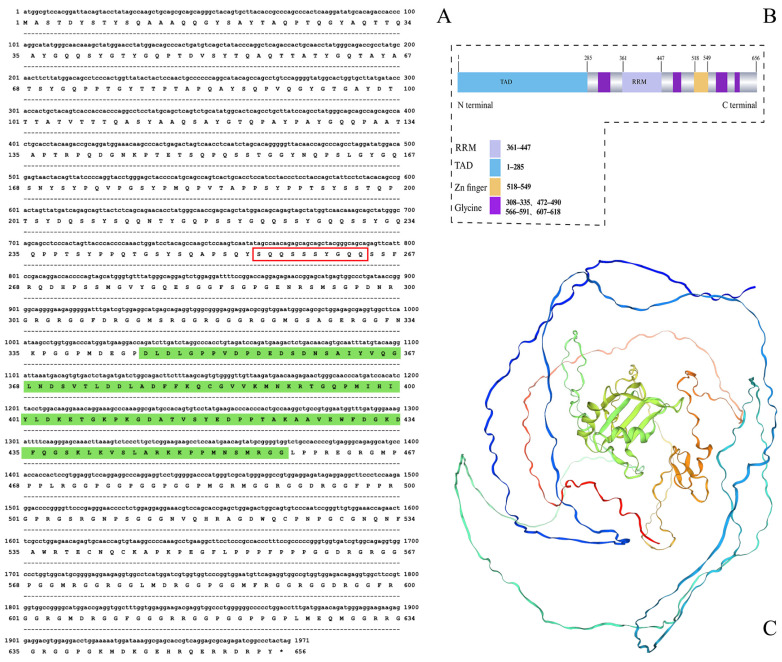
(**A**): Nucleotide sequence of the EWSR1 transcript (NM_005243) and corresponding amino acid sequence (NP_005234.1). * means that this codon does not encode an amino acid. (**B**): Schematic diagram of the EWSR1 domain structure. The frequent breakpoint sequence is highlighted in the red box. (**C**): Tertiary structure of EWSR1 predicted by AlphaFold (https://alphafoldserver.com/, accessed on 7 April 2026, clinical or research reference value is limited). The green segment corresponds to the experimentally resolved structure available in the PDB database, consistent with the green-labeled sequence in (**A**). (IBS 2.0 is used: an upgraded illustrator for the visualization of biological sequences). RRM: RNA recognition motif; TAD: Transactivation domain.

EWSR1 is a ubiquitously expressed RNA-binding protein in normal human cells that participates in the development of multiple tissues and organs as well as transcriptional and signaling regulation [47]. Its N-terminus contains a transactivation domain (TAD) enriched in serine, tyrosine, glycine, and glutamine residues. The C-terminus harbors an arginine-glycine-glycine (RGG) domain, and a central RNA recognition motif (RRM) is located between the TAD and RGG domain. Additionally, the *C*-terminal region contains numerous Arg-Gly-Gly (RGG) repeats that modulate RNA-binding activity. Studies have reported that approximately 75% (9/12) of EWSR1 breakpoints occur within the exon 7-encoded peptide sequence SQQSSSYGQQ. We show the nucleotide sequence of the EWSR1 transcript (NM_005243), the corresponding amino acid sequence (NP_005234.1), the domain structure, and the protein structure in Figure 3, which are deposited in the PDB (Figure 3) [46].

The EWSR1-WT1 fusion protein is generated by the fusion of the *N*-terminal transcriptional regulatory domain of EWSR1 to the *C*-terminal ZNF2–4 region of WT1. Anderson et al. [46] reported that the breakpoint sequences is SQQSSSYGQQ-SEKPYQCDFK. Owing to the retention of the spliceable region of WT1, two isoforms are produced: EWSR1-WT1(+KTS) and EWSR1-WT1(-KTS) [38,48,49]. Both isoforms participate in transcriptional regulation and share some common target genes while also regulating distinct sets of downstream targets [22,49,50,51]. Magrath et al. [22] observed that the ratio of EWSR1-WT1(+KTS) to EWSR1-WT1(-KTS) fluctuated between 1.3 and 1.7 in two DSRCT cell lines and one primary tumor. Although the former is more highly expressed, the latter exerts the major oncogenic effect. Consistent with the principle of KTS insertion in WT1, the insertion of KTS reduces the DNA-binding affinity of EWSR1-WT1, thereby altering the regulation of some downstream target genes. A study by Bandopadhayay et al. [51] also confirmed that the expression level of EWSR1-WT1(+KTS) is higher than that of EWSR1-WT1(-KTS), and most genes are regulated by the latter. So the vast majority of current DSRCT studies focus primarily on the EWSR1-WT1(-KTS) isoform.

To date, the high-resolution structure of the full-length EWSR1-WT1 fusion protein remains unresolved. Nevertheless, accumulating evidence has firmly established that EWSR1-WT1 can act directly or indirectly on multiple targets such as PDGFR, VEGFR, FGFR4 and IGF1R et al., thereby promoting tumorigenesis and development. These targets have become important breakthroughs for the treatment of DSRCT. The study aims to provide systematic evidence and feasible research directions for optimizing individualized treatment strategies and developing novel therapeutic targets for DSRCT.

## 4. Progress on Targeted Therapeutic

### 4.1. Receptor Tyrosine Kinase (RTK)

Multiple RTK inhibitors have demonstrated promising antitumor activity in the treatment of DSRCT. In a prospective study, 9 patients with DSRCT received sunitinib (*n* = 6), sorafenib (*n* = 2), with a median progression-free survival (mPFS) of 3.1 months [52]. In another study, disease control was achieved in 8 patients with DSRCT treated with sunitinib [53]. In 2018, Chen et al. [54] first reported the use of anlotinib, a multi-target tyrosine kinase inhibitor (TKI), in patients with post-surgical and post-chemotherapy progressive DSRCT, resulting in a 4-month PFS. Anlotinib simultaneously targets platelet-derived growth factor receptor (PDGFR), vascular endothelial growth factor receptor (VEGFR), mast/stem cell growth factor receptor (c-KIT), fibroblast growth factor receptor (FGFR), and other kinases. Jing et al. [55] further validated its efficacy in pediatric patients with DSRCT.

Pazopanib is another multi-targeted TKI that inhibits VEGFR, PDGFR, and c-KIT. In 2014, Frezza et al. [56] first reported the use of pazopanib in patients with metastatic DSRCT, with 7 of 9 patients achieving stable disease or partial response within 12 weeks. In a large retrospective study by Menegaz et al. including 26 patients with DSRCT, this disease control rate was approximately 62% (18/26) [57]. The efficacy of pazopanib has also been documented in other studies [58,59]. Collectively, the RTK family plays a pivotal role in DSRCT treatment. We summarize the clinical progress of major individual targets below.

#### 4.1.1. PDGF/PDGFR

PDGF and PDGFR are critical regulators of tumor stroma formation. PDGF acts on PDGFR to activate quiescent fibroblasts and smooth muscle cells, promoting DNA synthesis, cell proliferation, collagen matrix deposition, and neoangiogenesis [27,60,61]. Thus, the PDGF-PDGFR axis serves as a key driver of tumor progression [62]. PDGFA, an isoform of PDGF, is among the earliest identified downstream target genes of EWSR1-WT1. Lee et al. [61] demonstrated that PDGFA is highly expressed in the vast majority of DSRCT tissues and that EWSR1-WT1 directly induces PDGFA expression, thereby promoting desmoplasia. Similar observations were reported by Froberg et al., Negri et al., and Gerald et al. [38,63,64]. However, some studies have suggested an inverse correlation between PDGFA expression and the degree of desmoplasia in DSRCT [65].

PDGFR-α [66] and PDGFR-β [65] are the two major receptor isoforms, both of which are highly expressed in a subset of DSRCT tissues. In a clinical trial enrolling 2 patients with DSRCT, the small-molecule PDGFR inhibitor leflunomide (SU101) led to PFS exceeding 1 year in one patient [67]. Imatinib mesylate, a classic TKI that primarily targets PDGFR and c-KIT, is widely used for chronic myeloid leukemia. In a phase II clinical trial, among patients with DSRCT treated with imatinib mesylate, one patient with dual c-KIT- and PDGFR-α-positive disease achieved stable disease for 10 months, whereas another patient with PDGFR-α-negative tumors experienced disease progression within 1 month of treatment initiation [66]. A related clinical trial is currently ongoing (NCT00417807). Nonetheless, overall clinical outcomes of imatinib in DSRCT have been unsatisfactory [66,68,69].

In summary, the PDGF/PDGFR axis contributes significantly to DSRCT pathogenesis, and targeted therapies against this axis have achieved preliminary clinical benefits. However, therapeutic responses vary considerably across individuals, warranting further optimization and validation.

#### 4.1.2. VEGF/VEGFR

VEGF and its receptor VEGFR play essential roles in tumor angiogenesis. VEGF stimulates VEGFR signaling to promote angiogenesis, increase vascular permeability, and drive endothelial cell proliferation. The VEGF family comprises six members: VEGF-A, B, C, D, E, and placental growth factor (PlGF), among which VEGF-A exhibits the strongest angiogenic activity [70]. VEGFRs, which belong to the RTK family, are predominantly expressed on vascular endothelial cells and include three subtypes: VEGFR1, VEGFR2, and VEGFR3. VEGF signaling exerts pro-angiogenic effects mainly through VEGFR1 and VEGFR2, with VEGFR2 regarded as the primary functional receptor [71,72,73]. Targeting the VEGF/VEGFR axis has become a cornerstone of anticancer therapy and is also applicable to DSRCT [74].

Studies have shown that VEGFA and VEGFR2 are highly expressed in DSRCT cell lines and tumor tissues [75]. In DSRCT xenograft models, treatment with the VEGFA inhibitor bevacizumab resulted in favorable therapeutic outcomes. Given the VEGF dependence of DSRCT, several studies have reported significant efficacy with systemic chemotherapy combined with bevacizumab [75,76,77]. In a retrospective study [52], two patients with DSRCT treated with sorafenib, a multi-targeted TKI that primarily inhibits VEGFR2, achieved 3–4 months of PFS. Italiano et al. [53] reported the clinical activity of sunitinib, another TKI, in patients with DSRCT, with a median PFS of 2.6 months (95% CI: 0–9 months). Sunitinib exerts its anti-angiogenic effects by inhibiting multiple RTKs including VEGFR (especially VEGFR2) and PDGFR [78]. Apatinib is a classic oral small-molecule TKI that selectively targets VEGFR2. In 2018, Shi et al. [50] first reported its successful application in a patient with DSRCT. In 2020, Tian et al. [79] described a patient with DSRCT who achieved partial response following systemic chemotherapy with cyclophosphamide, epirubicin, and vincristine combined with apatinib. Additionally, a clinical trial investigating systemic chemotherapy plus ramucirumab, a VEGFR2 inhibitor, for DSRCT is currently underway (NCT04145349).

Although these studies provide promising therapeutic options for DSRCT, most are limited to case reports. The precise underlying mechanisms, safety profiles, and long-term efficacy require further systematic evaluation.

#### 4.1.3. FGFR4

The FGFR family consists of four members: FGFR1, FGFR2, FGFR3, and FGFR4. FGFR4 has been strongly implicated in the pathogenesis of pediatric embryonal rhabdomyosarcoma, and activated FGFR4 exhibits oncogenic activity [80]. In a study of 83 DSRCT tumor samples, Chow et al. [81] identified secondary genomic alterations of FGFR4 in approximately 82% of cases, highlighting its potential biological importance in DSRCT. Hingorani et al. [82] and Saito et al. [83] demonstrated that FGFR4 is a direct transcriptional target of the EWSR1-WT1 fusion gene, further supporting its pathogenic role in DSRCT. In a comprehensive analysis of 68 matched tumor-normal tissue pairs and 10 additional tumor specimens, Slotkin et al. identified FGFR4 as one of the key kinases dysregulated in DSRCT [84]. These findings support the development of FGFR4 inhibitors as a novel therapeutic strategy for patients with FGFR4-overexpressing DSRCT.

#### 4.1.4. IGF/IGF1R

Insulin-like growth factor 1 receptor (IGF1R) is a transmembrane RTK. It mediates the biological functions of insulin-like growth factors (IGF1 and IGF2) primarily through interactions with two signaling pathways: the RAS-rapidly accelerated fibrosarcoma-mitoge-activated protein kinase (RAS-RAF-MAPK) pathway and the phosphatidylinositol 3-kinase-protein kinase B/mammalian target of rapamycin (PI3K-PKB/Akt-mTOR) pathway [85,86]. Early studies documented frequent overexpression of IGF1R across multiple cancer types [87].

In 1993, Werner et al. demonstrated that wild-type WT1 partially represses IGF1R expression [88]. Given the structural and functional relationship between EWSR1-WT1 and wild-type WT1, subsequent studies investigated the regulatory effects of the fusion protein on IGF1R activity [89]. Notably, the two major isoforms of EWSR1-WT1 differentially modulate IGF1R transcription: EWSR1-WT1(−KTS) markedly enhances IGF1R promoter activity, whereas EWSR1-WT1(+KTS) does not. In 2002, Finkeltov et al. [90] confirmed that EWSR1-WT1 directly activates IGF1R expression, with isoform-specific differences in transactivation potency. Quantitative proteomic profiling further confirmed significant activation of IGF1R signaling in DSRCT [86]. Werner et al. [91] previously reported induction of IGF1R expression by an EWSR1-WT1 isoform in a 6-year-old male patient with DSRCT.

In 2020, Hingorani et al. [82] performed RNA sequencing on 12 EWSR1-WT1-positive DSRCT tumor tissues and found markedly elevated expression of IGF2, the ligand for IGF1R, in all samples. Further studies confirmed that IGF2 is a direct transcriptional target of EWSR1-WT1 and that IGF2 expression is significantly higher in DSRCT than in other sarcoma types, supporting robust activation of the IGF-IGF1R axis in this disease. Indeed, IGF2 has been implicated in the development and progression of numerous malignancies including breast cancer, Wilms’ tumor, and Ewing’s sarcoma [92].

Preclinical studies have shown that IGF1R-targeting monoclonal antibodies suppress vascular endothelial growth factor expression and attenuate AKT hyperphosphorylation induced by mTOR inhibitors in sarcoma models [93]. In a phase II clinical trial, 16 patients with DSRCT were treated with ganitumab, an anti-IGF1R monoclonal antibody, yielding an overall disease control rate (complete response + partial response + stable disease) of 63%, with a median PFS of 19 months (95% CI: 8.3–32.4 months) [94]. In another study [95], 3 patients with DSRCT received combination therapy with cixutumumab (anti-IGF1R antibody) and temsirolimus (mTOR inhibitor), resulting in durable PFS exceeding 5 months in 2 patients.

These findings provide strong preclinical and clinical evidence supporting the IGF/IGF1R axis as an actionable therapeutic target in DSRCT, suggesting that IGF1R inhibitors may represent a valuable treatment strategy.

#### 4.1.5. ERBB

The ERBB family, also known as the human epidermal growth factor receptor (HER) family, belongs to the RTK superfamily and comprises four members: EGFR (ErbB1/HER1), HER2 (ErbB2), HER3 (ErbB3), and HER4 (ErbB4) [96]. Several studies have reported HER2 expression in a subset of DSRCT tumors, with an expression rate of 92–100% [97,98,99]. Zhang et al. demonstrated that HER2-targeted antibody–drug conjugates (ADCs) exert potent antitumor activity in DSRCT patient-derived xenografts (PDX), cell lines, and organoid models. Brahmi et al. treated three HER2-positive patients with trastuzumab deruxtecan (T-DXd), all of whom achieved durable responses lasting more than 3 months [99]. Similar efficacy was recently reported by Renner et al. [100]. Furthermore, HER2-targeted bispecific antibody-based cellular immunotherapy has shown favorable antitumor activity in vitro and in xenograft models [97].

Smith et al. [101] demonstrated upregulation of multiple ERBB ligands, including EGF, amphiregulin, and epiregulin, in DSRCT. EGFR antagonists, such as cetuximab or small-molecule inhibitors, suppressed tumor cell growth in DSRCT cell lines, murine models, and PDXs, likely through inhibition of downstream RAS-RAF-MAPK-ERK and PI3K-AKT signaling. Notably, EGFR itself is not transcriptionally regulated by EWSR1-WT1. These findings offer new hope for patients with DSRCT and expand the landscape of targeted therapy for this disease.

#### 4.1.6. c-KIT (CD117)

The proto-oncogene c-KIT, also known as CD117, is overexpressed in a subset of patients with DSRCT [102,103]. In the report by Fine et al., the rate of IHC positive (≥2+) was 35%. Hingorani et al. [82] identified c-KIT as one of the most highly expressed surface markers in DSRCT, alongside CD200 and B7H3. As noted earlier, multiple TKIs including sunitinib, pazopanib, and apatinib exert antitumor effects partly through concurrent inhibition of c-KIT [78]. However, the overall positive rate of c-KIT expression in DSRCT remains relatively low [104,105,106]. For instance, Zhang et al. reported a c-KIT positivity rate of only 14%. Therefore, further investigation is warranted to fully define the therapeutic potential of c-KIT as a target in DSRCT.

#### 4.1.7. NTRK3

Neurotrophic tyrosine kinase receptor (NTRK) family members belong to the RTK superfamily and are implicated in the tumorigenesis of diverse human malignancies [107]. Among them, NTRK3 is a direct downstream target of EWSR1-WT1. Entrectinib, a selective inhibitor targeting NTRK3, has demonstrated significant antitumor activity in preclinical models of DSRCT [22,108]. A clinical trial evaluating NTRK-targeted therapy in DSRCT is currently ongoing (NCT04901806).

#### 4.1.8. MERTK

MER proto-oncogene, tyrosine kinase (MERTK) is an RTK implicated in the pathogenesis of multiple malignancies including rhabdomyosarcoma and represents a clinically actionable therapeutic target [48]. Bleijs et al. first reported that MERTK plays a critical oncogenic role in DSRCT in their study. The research team established a patient-derived OV-054 DSRCT in vitro model. Through RNA sequencing analysis of differentially expressed genes after EWSR1-WT1 knockdown, MERTK was found to be one of the most significantly downregulated genes, thereby initially identifying MERTK as a potential target regulated by EWSR1-WT1. To verify the role of MERTK, the study selected the MERTK small-molecule inhibitor UNC2025 and applied it to two DSRCT cell lines, OV-054 and JN-DSRCT-1, respectively. The results showed that UNC2025 significantly inhibited the proliferation of both DSRCT cell lines in a dose-dependent manner. In addition, the authors found through medium-throughput drug screening that this in vitro model is sensitive to a variety of drugs targeting apoptosis-related factors and RTK-mediated signaling pathways, suggesting that these two pathways are crucial in the development of DSRCT. Inhibiting MERTK can block multiple downstream signaling pathways mediated by it, including MAPK/ERK, PI3K/AKT, and JAK/STAT, which may be the core mechanism by which UNC2025 inhibits DSRCT cell proliferation.

### 4.2. mTOR

The PI3K-AKT-mTOR pathway is a canonical signaling cascade that has been widely implicated in the progression of various sarcomas [109,110]. A quantitative proteomic study revealed that the PI3K-AKT-mTOR pathway is activated in DSRCT, with mTOR complex 2 (mTORC2) as its primary effector [86]. Furthermore, Jiang et al. [26] reported a somatic mutation in the PIK3CA gene—which encodes the PI3K protein—in a patient with DSRCT. Collectively, these findings support a functional role of the PI3K-AKT-mTOR pathway in the development and progression of DSRCT.

An early preclinical study by Tirado et al. demonstrated that the mTOR inhibitor rapamycin induces apoptosis in DSRCT cell lines in vitro [111]. However, Dimitrakopoulou-Strauss et al. [112] observed limited efficacy of everolimus, a rapamycin derivative, in the treatment of DSRCT. In a clinical case of temsirolimus, another mTOR inhibitor, a patient with advanced DSRCT with progressive disease following multiple lines of chemotherapy and antiandrogen therapy achieved approximately 40 weeks of stable disease [113]. Wu et al. [114] showed that combined treatment with the PI3K inhibitor alpelisib and the mTOR inhibitor temsirolimus effectively suppressed DSRCT cell growth. Tarek et al. [115] administered a vinorelbine, cyclophosphamide, and temsirolimus (VCT) regimen to five patients with recurrent DSRCT, reporting partial responses in all subjects, with a median PFS of 8.5 months. In a retrospective study by Katz et al. [116], a patient with DSRCT who progressed on pazopanib monotherapy achieved 11 months of stable disease after receiving combination therapy with pazopanib and the mTOR inhibitor sirolimus. These observations suggest that mTOR inhibitors may yield superior therapeutic efficacy in combination with other agents compared with monotherapy. Moreover, as IGF1R functions as an upstream regulator of the PI3K-AKT-mTOR pathway, several investigational studies have combined IGF1R-targeted monoclonal antibodies with mTOR inhibitors and achieved promising clinical activity [95].

### 4.3. Androgen Receptors

The striking male predominance of DSRCT has long attracted research interest, and subsequent investigations have revealed a high positive expression rate of the androgen receptor (AR) in DSRCT specimens [10,15,103,117]. The AR positive rate reported by Fine et al. is 37% for IHC ≥ 2+ [103]. And Bulbul et al. [15] demonstrated that dihydrotestosterone (DHT) stimulates DSRCT cell proliferation, an effect that can be abrogated by AR antagonists. In the study by Fine et al. [103], 3 of 6 patients with DSRCT who received androgen deprivation therapy achieved disease remission lasting 3 to 6 months. Similarly, Lamhamedi-Cherradi et al. [118] reported that the AR antagonist enzalutamide and AR-targeted antisense oligonucleotides (AR-ASO) effectively blocked DHT-induced DSRCT cell proliferation and markedly reduced tumor burden in xenograft models. These authors proposed that DSRCT represents the third androgen-driven malignancy, following prostate cancer and AR-positive triple-negative breast cancer [119], and that AR-targeted therapy may represent a novel therapeutic strategy. Gedminas et al. [25] further noted that the EWSR1-WT1 fusion protein represses estrogen-related signaling, consistent with the androgen dependence of DSRCT. However, a recent study [19] challenged this paradigm by demonstrating that androgens and AR are dispensable for the in vitro growth of DSRCT cells, despite the marked male predominance of the disease and the growth-inhibitory effects of AR antagonists such as enzalutamide and flutamide. The authors hypothesized that the male predominance of DSRCT may be attributed to a role of androgens in promoting the chromosomal translocation that generates the EWSR1-WT1 fusion, rather than being required for sustained tumor growth.

Supporting this notion, several studies have documented that AR can physically interact with EWSR1 [120] and WT1 [118] under certain conditions, thereby potentially influencing chromosomal breakage and rearrangement. Analogous interactions between AR and fusion-related proteins have been reported in specific subtypes of prostate cancer, lending further credence to this hypothesis. [121]. Other studies have suggested that the growth-inhibitory effects of enzalutamide in DSRCT may be mediated indirectly through the glucocorticoid receptor (GR, encoded by NR3C1), a mechanism also observed in prostate cancer. [122]. Furthermore, AR-positive DSRCT cells have been reported to exhibit enhanced stem-like properties, which may contribute to tumor heterogeneity and limit the efficacy of AR-targeted therapies.

Nevertheless, the precise molecular mechanisms underlying AR function in DSRCT pathogenesis and progression remain incompletely defined, and the clinical efficacy of AR-targeted therapies requires validation in larger prospective cohorts. Future therapeutic directions may include AR inhibitor monotherapy or rational combinations with chemotherapy to improve response rates and survival outcomes for patients with DSRCT.

### 4.4. B7H3 (CD276)

The immunomodulatory protein B7 homolog 3 (B7H3, also known as CD276) is overexpressed in a wide spectrum of malignancies and correlates with poor overall survival [123]. In the meta-analysis including 4623 patients by Zhang et al., the positive rate of B7H3 was approximately 69%. Targeting B7H3 has been shown to suppress tumor growth and progression [124]. Multiple studies have reported high expression of B7H3 (CD276) in DSRCT, supporting its potential as a therapeutic target [82,125,126]. A clinical trial investigating enoblituzumab (MGA276), a monoclonal antibody targeting B7H3, for the treatment of solid tumors including DSRCT is currently underway (ClinicalTrials.gov identifier: NCT02982941). Current therapeutic development targeting B7H3 mainly focuses on B7H3-directed radioimmunotherapy and cellular immunotherapy (discussed below).

### 4.5. CDK4/6

Cyclin-dependent kinase 4/6 (CDK4/6) are serine/threonine kinases activated by cyclin D, which in turn phosphorylate the retinoblastoma-associated protein (RB) to drive cell cycle progression from the G1 phase to the S phase [107]. Magrath et al. [22] demonstrated that EWSR1-WT1(−KTS) promotes tumorigenesis via the CCND–CDK4/6–RB axis. Pharmacological inhibition of CDK4/6 using agents such as palbociclib, or genetic silencing of RB, markedly suppresses DSRCT cell growth. These findings were independently validated by Boulay et al. [49]. Given that estrogen signaling can activate this pathway and has shown therapeutic efficacy in breast cancer, the authors proposed that CDK4/6 inhibitors combined with anti-estrogen therapy represents a rational therapeutic strategy for DSRCT [127]. Of note, the CCND–CDK4/6–RB axis is also aberrantly activated in Ewing sarcoma. In a related clinical trial, combination treatment with the CDK4/6 inhibitor palbociclib and the IGF1R inhibitor ganitumab resulted in approximately 30% of patients achieving PFS at 6 months [128].

### 4.6. SIK1

Salt-inducible kinase 1 (SIK1) is another direct downstream target of EWSR1-WT1 that is significantly upregulated in DSRCT [129,130]. Pharmacological inhibition or genetic silencing of SIK1 effectively suppresses DSRCT tumor growth. A proposed mechanism is that SIK1 inhibition reduces the activity of minichromosome maintenance protein 2 (MCM2), a key regulator of DNA replication initiation [107].

### 4.7. Other Targets

Through sequencing analysis, Mello et al. [131] identified 15 somatically mutated genes in DSRCT, of which 7 were regulated by lymphoid enhancer-binding factor 1 (LEF1), a known downstream target of WT1. These findings suggest that LEF1 may participate in DSRCT pathogenesis, although its precise molecular mechanism remains to be elucidated. Recent studies have further revealed that a set of neogenes, which are transcriptionally silent in normal tissues, can be aberrantly activated by EWSR1-WT1 in DSRCT, representing a novel class of potential therapeutic targets [107,132]. Geyer et al. [20] reported that calcium voltage-gated channel auxiliary subunit alpha2delta 2 is highly and specifically expressed in DSRCT, supporting its utility as a promising diagnostic biomarker and therapeutic target. Previous studies [38] have demonstrated that EWSR1-WT1(-KTS) partially exerts its oncogenic functions via the interleukin-2 receptor β (IL-2Rβ)–STAT3 signaling axis. In addition, several genes are specifically upregulated by the fusion protein, including BAIAP3, myelodysplasia/myeloid leukemia factor 1 (MLF1), and T-cell acute lymphoblastic leukemia-associated antigen (TALLA-1). By contrast, leucine-rich repeat-containing protein 15 (LRRC15) may be preferentially regulated by EWSR1-WT1(+KTS). Somatostatin receptors (SSTRs) have recently been found to be overexpressed in DSRCT [100,133]. A clinical trial is currently evaluating the efficacy of pasireotide, a long-acting somatostatin analog, as maintenance therapy in patients with synovial sarcoma and DSRCT (NCT06456359) [133]. A phase 2 trial targeting dopamine receptor D2 (DRD2) showed clinical benefit in patients with DSRCT (NCT03034200) [134]. Magrath et al. [135] reported that B-lymphoid kinase (BLK) is transcriptionally upregulated by EWSR1-WT1, and treatment with dasatinib and other kinase inhibitors effectively suppressed DSRCT progression. However, these results were not recapitulated in in vitro studies by van Erp et al. [136]. Hartlapp et al. [137] administered CXCR4-directed [^90^Y] peptide receptor radionuclide therapy to four patients with DSRCT. Among them, two patients achieved durable stable disease for 143 days and 176 days, respectively, and one patient achieved a partial response.

Table 1 summarizes the main drugs, corresponding targets, evidence and other information related to targeted therapy.

## 5. Current Status and Advances in Immunotherapy

Sarcomas, including DSRCT, are generally insensitive to immunotherapy. However, as one of the important means of anti-tumor therapy, research into DSRCT immunotherapy has not ceased. This article will sort out DSRCT-related immunotherapy from the perspective of immunotherapeutic targets.

### 5.1. PD1-PDL1

Positive expression of programmed cell death protein 1 (PD-1) and programmed death ligand 1 (PD-L1) serves as an independent prognostic indicator of poor overall survival (OS). Although sarcomas are generally considered to have low tumor mutational burden and thus less amenable to immunotherapy, co-expression of PD-L1 on tumor cells and PD-1 on tumor-infiltrating lymphocytes has also been associated with unfavorable prognosis [138]. DSRCT is widely regarded as an immunologically cold tumor, typically lacking prominent PD-1 and PD-L1 expression [10,15,114,117]. However, another study examining tumor samples from 11 patients with DSRCT reported a high PD-1 positivity rate of 82%, of which approximately 73% showed strong expression. In contrast, the PD-L1 positivity rate was only about 18% [139]. Subsequent functional testing of nivolumab in PD-1-positive DSRCT cell lines showed limited antitumor activity. Similarly, Negri et al. [64] found that blockade of the PD-1/PD-L1 axis exerted no significant therapeutic effect in DSRCT models, which may be attributable to the low mutational burden of DSRCT. Nevertheless, recent studies have demonstrated clinical benefit from immunotherapy in selected soft tissue sarcomas, including DSRCT [131,140]. Schöffski et al. used Pembrolizumab plus Olaratumab in the treatment of patients with soft tissue sarcomas, and 6 of 28 patients achieved partial response, including 1 patient with DSRCT. The median PFS was 4.2 months and the median OS was 14.8 months, and the efficacy seemed to be unrelated to the PDL1 expression rate. A phase II clinical trial is currently evaluating the activity of the PD-1 inhibitor pembrolizumab in a subset of advanced sarcomas (ClinicalTrials.gov identifier: NCT02301039).

### 5.2. B7H3

The immunologically cold phenotype of DSRCT has hindered the broad application of conventional immunotherapy. However, precision delivery of cytotoxic immune cells [141] or radionuclides (radioimmunotherapy, RIT) [142] to tumor sites via targeting of cancer cell surface antigens has emerged as a promising therapeutic strategy, for which identification of reliable target antigens is critical. B7H3 was the first such target identified in DSRCT, characterized by uniform, widespread, and intense expression in this tumor. High expression of B7H3 in DSRCT therefore represents a promising target not only for targeted therapy but also for radioimmunotherapy and antigen-directed CAR-T cell therapy [82,97,107]. A phase I clinical trial enrolling 48 patients with DSRCT demonstrated that ^131^I-omburtamab, a B7H3-targeted radioimmunotherapeutic agent, exhibited favorable safety profiles and achieved effective control of intra-abdominal disease [143]. Study has shown that the efficacy of RIT in patients with measurable disease is better than those with no evaluable disease. Therefore, this therapy is more effective in patients who have undergone debulking surgery and achieved R1/R0 resection. A corresponding phase II trial is currently underway (ClinicalTrials.gov identifier: NCT04022213). In addition, two clinical trials investigating B7H3-directed CAR-T cell therapy for solid tumors (NCT04483778 and NCT04897321) are ongoing, with encouraging preliminary potential.

### 5.3. GD2

Ganglioside GD2 (GD2) is a tumor-associated surface antigen that is highly expressed in sarcomas affecting children, adolescents, and young adults [144]. It can induce tyrosine phosphorylation and activate multiple kinase signaling pathways, thereby enhancing the proliferation, migration, and invasion capabilities of cancer cells. Studies have demonstrated high GD2 expression in DSRCT [125], supporting its potential as a target for immunotherapy. Espinosa-Cotton et al. showed that GD2-targeted T cell-engaging bispecific antibodies exerted promising antitumor activity in vitro of DSRCT [97]. The authors also found that the cytotoxicity of the antibodies is related to the expression level of the corresponding targets on the cell surface. Additionally, the authors confirmed that other antigens, such as EGFR, HER2, and mesothelin, could also serve as targets for similar bispecific antibody-based therapies. A clinical trial by Yankelevich et al. [145] also preliminarily validated the efficacy of anti-CD3 + anti-GD2 bispecific antibody-armed T cells in the treatment of various sarcomas, including DSRCT. A total of nine patients were enrolled in this study. The only patient with DSRCT received merely three cycles of treatment due to rapid disease progression. The remaining eight patients completed all eight cycles of therapy, among whom five achieved an overall survival of more than one year. The median overall survival of all nine patients was 18.0 months. Therefore, full-cycle treatment may be correlated with better clinical outcomes. Further clinical evaluation involving more DSRCT patients is warranted in the future.

### 5.4. Other Immune Targets

Overexpression of CD200 [82,131] and mesothelin [97] has also been identified in DSRCT, indicating their potential as additional targets for immunotherapeutic strategies. Unfortunately, current research remains at the preclinical stage. It has been proposed that the amino acid sequence at the fusion junction of the EWSR1-WT1 fusion protein itself can serve as a novel peptide epitope recognized by immune cells, thereby functioning as an immunotherapeutic target. The concept has been validated in fusion protein-driven diseases such as chronic myelogenous leukemia [107].

Furthermore, the EWSR1-WT1 fusion event can lead to the generation of neogenes [146]. Many tumors produce abnormal transcription factors due to gene fusions, such as EWS-FLI1 in Ewing sarcoma and EWSR1-WT1 in DSRCT. These transcription factors are also called oncogenic chimeric transcription factors (OCTFs). In DSRCT, EWSR1-WT1 induces abnormal transcription of originally silent genomic regions in DSRCT cells through its unique transcriptional activation activity, thereby generating a type of tumor-specific novel transcript that constitutes the unique neogenes of DSRCT. The core mechanism is that the EWSR1-WT1 fusion protein specifically binds to specific regions in the genome through the DNA-binding ability of its WT1 domain, and simultaneously recruits RNA polymerase II and chromatin remodeling complexes to relieve the chromatin silencing state of this region and initiate the abnormal transcription process. The neotranscripts corresponding to these neogenes are mostly multi-exonic structures (accounting for approximately 58.7%), which are specifically expressed only in DSRCT cells, almost not expressed in normal tissues and other types of tumors, and their expression is completely dependent on the EWSR1-WT1 fusion protein. The transcriptional activation of some neogenes is also related to the enhancer-promoter regulatory chains mediated by EWSR1-WT1. The fusion protein binds to the enhancer region in the regulatory chain and regulates the transcription of neogenes at a long distance through changes in chromatin conformation. In conclusion, the EWSR1-WT1 fusion event generates neogenes with DSRCT specificity and fusion protein-dependent regulation by abnormally activating silent genomic regions. These neogenes are not only one of the molecular characteristics of DSRCT, but some can also be translated into tumor-specific neopeptides, providing potential targets for the diagnosis and immunotherapy of DSRCT. Vibert et al. [146] and Magrath et al. [107] identified 37 novel neogenes in DSRCT, the majority of which showed stable expression. These neogenes provide new potential avenues for the development of targeted immunotherapies for DSRCT.

In summary, there is no valid evidence to prove the effectiveness and effective conditions of traditional immune checkpoint inhibitors in the treatment of DSRCT, and further exploration is still needed in the future. Promisingly, radioimmunotherapy and cellular immunotherapy targeting tumor surface-associated antigens are in a stage of rapid development, and B7H3 seems to be one of the most researched targets at present, which is worthy of expectation.

Table 2 summarizes the main drugs, corresponding targets, evidence and other information related to immunotherapy.

## 6. DNA Damage Response (DDR)

Under the influence of internal and external factors, a large number of DNA damages occur in the human body every day. These damages trigger a series of signaling pathways to respond to DNA lesions, collectively referred to as the DDR [147]. DDR plays a crucial role in the initiation and progression of tumor cells. The poly(ADP-ribose) polymerase (PARP) family is a key component of the DDR network. In recent years, PARP inhibitors (PARPis) have demonstrated promising efficacy in antitumor therapy, particularly in tumors with homologous recombination repair deficiencies caused by mutations in genes such as BRCA [148]. A genomic analysis revealed that among 135 identified mutated genes, approximately 27% are associated with the DDR network and mesenchymal-epithelial transition [149]. In another gene sequencing study, some secondary genetic mutations in a subset of DSRCT were linked to DDR [81].

In a study by Van Erp et al., PARP1—the most abundant enzyme in the PARP family—was upregulated in all DSRCT tissues, and the DNA damage repair marker schlafen-11 (SLFN11) was overexpressed in 92% of tissues. Further investigations showed that the combination of the PARP inhibitor olaparib and the alkylating agent temozolomide achieved promising antitumor effects in both in vitro and in vivo experiments [150]. Similarly, Mellado-Lagarde et al. confirmed SLFN11 expression in DSRCT and demonstrated that PARPi monotherapy or its combination with irinotecan or ionizing radiation exerted favorable efficacy in preclinical models [151]. Trabectedin is a chemotherapeutic agent that promotes DNA damage and inhibits its repair [152]. The combination of trabectedin and the PARP inhibitor olaparib achieved encouraging results in a preclinical mouse model of sarcoma [153] and a phase IB clinical trial of sarcoma [154]. Another clinical trial [155] evaluating trabectedin for the treatment of various rare sarcomas showed that 1 out of 3 DSRCT patients achieved complete response [146]. Therefore, the application of trabectedin alone or in combination with PARP inhibitors such as olaparib in DSRCT merits further investigation.

Checkpoint kinase 1 (CHK1) is an important molecule involved in DDR. Lowery et al. [156] preliminarily validated the efficacy of the CHK1 inhibitor prexasertib in two DSRCT xenograft models. In a subsequent clinical trial, prexasertib in combination with irinotecan achieved a disease control rate of 79% in 21 solid tumor patients, including 19 with DSRCT [157]. In summary, DDR plays a vital role in the development and progression of DSRCT, and new breakthroughs are anticipated in future research and clinical applications.

Table 3 summarizes the main drugs and other information related to DDR.

## 7. Discussion

DSRCT is an extremely rare malignant soft tissue neoplasm with an extremely poor prognosis, predominantly affecting adolescent and young adult males aged 20–30 years. Due to its non-specific clinical and pathological features and low incidence, DSRCT is often diagnosed at an advanced stage, with a relatively short research history [4]. The fundamental pathogenic mechanism of DSRCT is the reciprocal chromosomal translocation t(11;22)(p13;q12)—a finding first reported by Sawyer et al. in 1992 [34]. Consequently, genetic testing for the EWSR1-WT1 fusion gene has become the gold standard for DSRCT diagnosis. However, recent studies have documented rare cases of EWSR1-WT1 expression in non-DSRCT tumors, highlighting the need for careful differential diagnosis [33].

Since its initial identification, research on DSRCT has continued unabated, but no consensus or clinical guidelines for DSRCT treatment have been established to date. Surgery remains the most effective therapeutic modality for DSRCT and can significantly improve patient prognosis [158,159]. However, due to extensive intra-abdominal seeding and peritoneal metastasis, complete surgical resection is often unachievable, and microscopic residual lesions may remain postoperatively. Therefore, multimodal comprehensive treatment combining surgery with hyperthermic intraperitoneal chemotherapy (HIPEC) [160], systemic chemotherapy, targeted therapy, and radiotherapy is necessary. Osborne et al. [161] evaluated the efficacy of cytoreductive surgery (CRS) + HIPEC combined with WAPT, confirming a significant improvement in 5-year survival rates. Hayes-Jordan et al. [162] performed CRS+HIPEC in 14 patients with DSRCT, achieving a median overall survival (OS) of 44.3 months, a median recurrence-free survival (RFS) of 14.0 months, and a 3-year OS of 79% from the time of diagnosis. A recent study demonstrated that even 9 patients who only achieved R2 resection following CRS still derived clinical benefit from surgery [18]. However, the complications associated with major surgery and HIPEC cannot be ignored. A retrospective study of 9 DSRCT patients who underwent CRS+HIPEC reported high postoperative recurrence rates, with long-term parenteral nutrition required in some cases due to impaired gastrointestinal function; gastrointestinal complications such as partial intestinal obstruction and genitourinary complications may even necessitate reoperation [163].

Targeted therapy, the focus of this review, is an indispensable component of DSRCT treatment, most commonly used in combination with chemotherapy. Studies have consistently shown elevated expression of PDGF/PDGFR, VEGFR, FGFR4, IGF/IGF1R, HER2, c-KIT in DSRCT [82,86,97,98,103]. This upregulation is partially attributed to the loss of WT1-mediated transcriptional repression following EWSR1-WT1 fusion [164], leading to the activation of approximately 35 downstream target genes [165,166]. Since these genes are either RTKs or their ligands, targeted drugs such as imatinib mesylate, anlotinib, sunitinib, pazopanib, apatinib, ganitumab, bevacizumab, and cetuximab have been used clinically in patients with positive expression of specific targets. Unfortunately, while some studies have reported prolonged disease-free survival or disease remission, others have failed to achieve expected therapeutic effects. This may be partly due to low target gene expression levels and partly to the development of target gene resistance. Further research is needed to optimize the development and application of RTK-related targets.

The mTOR, AR have also provided additional therapeutic options for patients with advanced DSRCT. The use of mTOR inhibitors such as temsirolimus alone [113] or in combination with other targeted agents such as pazopanib [116] and IGF1R monoclonal antibodies [95] has been shown to prolong survival in DSRCT patients. Drawing on the experience of two other AR-driven malignancies (prostate cancer and AR-positive triple-negative breast cancer) [119], studies have demonstrated that AR antagonists such as enzalutamide and flutamide can inhibit the in vitro growth of DSRCT [19], with further clinical investigations underway. Additionally, CDK4/6 inhibitors such as palbociclib, which block the CCND–CDK4/6–RB axis, represent a promising therapeutic strategy [49].

DSRCT is generally insensitive to immunotherapy, primarily due to its low tumor mutational burden (TMB) [15,64]—a well-established predictor of poor response to immune checkpoint inhibition, as lower TMB is typically associated with inferior immunotherapeutic efficacy [167,168]. Consistent with this, studies have confirmed low positive expression rates of PD-1 and PD-L1 in DSRCT [10,15,117], with unsatisfactory outcomes of PD-1/PD-L1 pathway blockade [64,139]. However, research into DSRCT immunotherapy has not ceased. Given the high expression of immunomodulatory molecules such as B7H3 [82,125,126] and GD2 [125] in DSRCT, novel immunotherapeutic strategies have been developed, including B7H3-directed intraperitoneal radioimmunotherapy with ^131^I-8H9 (NCT01099644) and GD2-directed bispecific antibody-based cellular immunotherapy [97]. The discovery of additional immunotherapeutic targets is eagerly anticipated to expand treatment options for DSRCT.

Emerging evidence suggests that DDR may be involved in DSRCT pathogenesis: genetic sequencing studies have identified DDR-related gene mutations in DSRCT [81,149], and elevated expression of SLFN11—a key DDR marker—has also been reported. Further preclinical and clinical studies have demonstrated promising efficacy of PARPis alone, in combination with chemotherapy, or with the DNA-damaging agent trabectedin in the treatment of DSRCT, highlighting the potential of DDR-targeted therapies as a novel treatment direction for this disease.

Clinical trials represent a high-level evidence approach, and there is an urgent desire to identify effective therapeutic methods through high-level evidence. This review also covers relevant clinical trials, which are summarized in Table 4.

## 8. Future Perspectives

In conclusion, in terms of targeted therapy, TKIs remain an important component of DSRCT treatment, and drugs targeting different molecular targets can complement each other. Among these targets, precise targeted therapy against HER2 has attracted increasing attention in recent years. Downstream targets regulated by EWSR1-WT1, such as NTRK3 and MERTK, have initially shown potential therapeutic value in preclinical studies, which still require further in-depth research. As a gender-differentiated tumor, the mechanism of action of the AR in DSRCT and the corresponding targeted therapy have long been a focus of attention, and we have reason to believe that key breakthroughs will be achieved in this field in the future. In the field of immunotherapy, as a “cold tumor”, DSRCT has encountered bottlenecks in the application of traditional immune checkpoint inhibitors. However, radioimmunotherapy and cellular immunotherapy targeting B7H3, HER2, and GD2 are gradually gaining attention, with multiple clinical trials focusing on these strategies. In addition, neogenes and neopeptides induced by EWSR1-WT1, as tumor-specific antigens, hold great prospects for immunotherapy. Finally, the DDR is an important participant in the occurrence of DSRCT; the significant upregulation of markers such as PARP1, SLFN11, and CHK1 may indicate that drugs inhibiting DDR, such as PARPi and trabectedin, can exert certain therapeutic effects.

Despite significant progress in understanding the pathogenesis and therapeutic strategies of DSRCT, challenges remain, and the path ahead is not smooth. Future research should focus on clarifying the precise molecular mechanisms of EWSR1-WT1-mediated tumorigenesis, identifying novel actionable targets, optimizing multimodal treatment regimens, and conducting large-scale prospective clinical trials to improve the prognosis of patients with this devastating disease.

## Figures and Tables

**Table 1:** Summary of main drugs and targets for targeted therapy.

Representative Agents	Relevant Targets	References	Evidence Type	Trial IDs	Phase	DSRCT Sample	Outcomes
leflunomide (SU101)	PDGFR	Adamson et al. [67]	clinical trial	NCT00001573	I	2	PFS exceeding 1 year in one patient
imatinib mesylate	PDGFR, c-KIT	Chao et al. [66]	clinical trial	NCT00062205	II	-	one patient achieved stable disease for 10 months
sunitinib	VEGFR, PDGFR	Italiano et al. [53]	retrospective study	-	-	8	median PFS of 2.6 months
		Bétrian et al. [52]	retrospective study			6	median PFS was 3.1 months
sorafenib	VEGFR, PDGFR	Bétrian et al. [52]	retrospective study	-	-	2	two patients achieved 3–4 months of PFS
bevacizumab	VEGF	Magnan et al. [76]	preclinical study	-	-	-	irinotecan combined with bevacizumab has a more significant inhibitory effect on xenografts than irinotecan alone.
anlotinib	PDGFR, VEGFR, c-KIT, FGFR	Chen et al. [54]	case report	-	-	1	after 5 cycles of anlotinib, the tumor lesions showed progressive shrinkage.
		Jing et al. [55]	prospective trial	-	-	3	anlotinib is effective in children with DSRCT.
pazopanib	VEGFR, PDGFR, and c-KIT	Frezza et al. [56]	retrospective study	-	-	9	7 of 9 patients achieving stable disease or partial response within 12 weeks
		Menegaz et al. [57]				29	disease control rate was approximately 62%
apatinib	VEGFR	Shi et al. [50]	case report	-	-	1	partial remission
		Tian et al. [79]				1	achieved disease remission with combined systemic chemotherapy
ramucirumab	VEGFR	-	clinical trial	NCT04145349	I/II	30	unknown
NA	FGFR4	Chow et al. [81] Slotkin et al. [84]	preclinical study	-	-	-	potential therapeutic targets
ganitumab	IGF1R	Tap et al. [94]	clinical trial	NCT00563680	phase II	16	fifty-five percent of patients achieved remission or stable disease
cixutumumab		Naing et al. [95]	retrospective study	-	-	3	2 patients had stable disease
trastuzumab deruxtecan	HER2	Brahmi et al. [99]	case report	-	-	3	showed a notable activity in all patients
cetuximab	EGFR	Smith et al. [101]	preclinical study	-	-	-	potent inhibitory effects on tumor cells and xenografts
entrectinib	NTRK3	Ogura et al. [108]	preclinical study	-	-	-	significantly reduces growth of DSRCT cells both in vitro and in vivo
UNC2025	MERTK	Bleijs et al. [48]	preclinical study	-	-	-	Significantly inhibits the proliferation of tumor cells
temsirolimus	mTOR	Thijs et al. [113]	case report	-	-	-	achieved a 40-week disease stabilization
		Wu et al. [114]	preclinical study				combined with PI3K inhibitor to inhibit cell proliferation
		Tarek et al. [115]	case report			5	combined with VCT chemotherapy, with a median PFS of 8.5 months
rapamycin		Tirado et al. [111]	preclinical study	-	-	-	induced the apoptotic death of cells
enzalutamide	AR	Lamhamedi-Cherradi et al. [118]	preclinical study	-	-	-	inhibit cell proliferation and reduce xenograft tumor burden
		Fine et al. [103]	prospective trial			6	three patients achieved disease stabilization for 3–4 months.
enoblituzumab	B7H3	-	clinical trial	NCT02982941	I	-	unknown
palbociclib	CDK4/6	Magrath et al. [22] Boulay et al. [49]	preclinical study	-	-	-	reduced growth in DSRCT xenograft models
YKL-05-099	SIK1	Hartono et al. [129]	preclinical study	-	-	-	inhibition of tumor cell growth

PFS: progression free survival; DSRCT: desmoplastic small round cell tumor; VCT: vinorelbine, cyclophosphamide, and temsirolimus.

**Table 2:** Summary of main drugs and targets for immunotherapy.

Representative Agents	Relevant Targets	References	Evidence Type	Trial IDs	Phase	DSRCT Sample	Outcomes
pembrolizumab	PD1	-	clinical trial	NCT02301039	II	0	unknown
I^131^-Omburtamab	B7H3	Modak et al. [143]	clinical trial	NCT01099644	I	-	safe and effective
		-		NCT04022213	II	-	unknown
CAR T Cell Immunotherapy		-	clinical trial	NCT04483778	I	-	unknown
				NCT04897321	I	-	unknown
GD2 bispecific antibody	GD2	Espinosa-Cotton et al. [97]	preclinical study	-	-	-	showed cytotoxicity
		Yankelevich et al. [145]	clinical trial	NCT02173093	I	1	cannot be evaluated
bispecific antibody	EGFR, HER2, and mesothelin	Espinosa-Cotton et al. [97]	preclinical study	-	-	-	showed cytotoxicity

**Table 3:** Summary of main drugs and key information related to DDR.

Representative Agents	References	Evidence Type	Trial IDs	Phase	DSRCT Sample	Outcomes
olaparib	van Erp et al. [150]	preclinical study	-	-	-	reduced cell viability and cell migration in vitro
	Grignani et al. [154]	clinical trial	NCT02398058	I	-	unknown
PARPi	Mellado-Lagarde et al. [151]	preclinical study	-	-	-	DSRCT is sensitive to PARPi combination therapy
Trabectedin	Aune et al. [152]	preclinical study	-	-	-	induce DNA damage
	Pignochino et al. [153]	preclinical study	-	-	-	PARP1 inhibition potentiated trabectedin activity
	Grignani et al. [154]	clinical trial	NCT02398058	I	-	unknown
	Palmerini et al. [155]	clinical trial; retrospective study	NCT02793050	-	3	6-months PFS was 33%
prexasertib	Lowery et al. [156]	preclinical study	-	-	-	has significant anti-tumor effects
	Slotkin et al. [157]	clinical trial	NCT04095221	I/II	19	prexasertib in combination with irinoteca n is promising

DSRCT: desmoplastic small round cell tumor; DDR: DNA damage response; PFS: progression free survival; PARPi: poly ADP-ribose polymerase inhibitors.

**Table 4:** Summary of clinical trial.

**Trial Number**	**Phase**	**Relevant Targets**	**Treatment**	**Sponsor**	**Disease Indications**
NCT00001573	I	PDGFR	SU101	National Cancer Institute	Glioma, Sarcoma
NCT00062205	II	PDGFR; c-KIT	imatinib mesylate	City of Hope Medical Center	Sarcoma
NCT00417807	II	PDGFR; c-KIT	imatinib mesylate	Novartis Pharmaceuticals	DSRCT
NCT04145349	I/II,	VEGFR	ramucirumab	Eli Lilly and Company	DSRCT
NCT00563680	II	IGF1R	Ganitumab	NantCell, Inc.	Ewing’s Family Tumor, DSRCT
NCT04901806	I	NTRK	PBI-200	Pyramid Biosciences	Advanced or metastatic solid tumors
NCT02982941	I	*B7H3*	Enoblituzumab	MacroGenics	Solid Tumors
NCT06456359	II	SSTR	Pasireotide	University Hospital Heidelberg	DSRCT and Synovial sarcoma
NCT03034200	II	dopamine receptor D2	ONC201	Peter Anderson	Neuroendocrine cancers
NCT02301039	II	PD-1	pembrolizumab	Sarcoma Alliance for Research through Collaboration	Bone Sarcoma Soft Tissue Sarcoma
NCT01099644	I	B7H3	B7H3-targeted radioimmunotherapeutic	Y-mAbs Therapeutics	Peritoneal Cancer
NCT04022213	II		I^131^-Omburtamab	Memorial Sloan Kettering Cancer Center	DSRCT
NCT04483778	I		B7-H3 CAR T cells	Seattle Children’s Hospital	Recurrent/Refractory Solid Tumors
NCT04897321	I		B7-H3 CAR T cells	St. Jude Children’s Research Hospital	Solid Tumors
NCT02173093	I	GD2	GD2 bispecific antibody	University of Virginia	Neuroblastoma and Osteosarcoma
NCT02398058	IB	DDR	Trabectedin and olaparib	Italian Sarcoma Group	Soft Tissue Sarcoma
NCT02793050	retrospective	DDR	Trabectedin	Italian Sarcoma Group	Sarcoma, Soft Tissue
NCT04095221	I/II	DDR	Trabectedin, Irinotecan	Memorial Sloan Kettering Cancer Center	DSRCT, Rhabdomyosarcoma

DSRCT: desmoplastic small round cell tumor; DDR: DNA damage response; CAR: chimeric antigen receptor.

## Data Availability

No new data were created or analyzed in this study.
